# The Liverpool Psycho-Therapeutic Institution

**Published:** 1914-02-28

**Authors:** Sidney Wilkinson

**Affiliations:** one of the Honorary Physicians


### THE LIVERPOOL PSYCHO-THERAPEUTIC INSTITUTION.

By SIDNEY WILKINSON, M.R.C.S., L.R.C.P., one of the Honorary Physicians.

A significant event in the history of English psycho-therapy was marked on Monday, November 11, 1912, when the doors of No. 8 Maryland Street, Liverpool, were opened to a little group of waiting patients, for thus commenced the first charitable institution in this country for the treatment of functional diseases by suggestion. The want had been realised for a long time by those members of the Liverpool Psycho-Medical Society who are engaged in this branch of treatment. A committee was formed and subscriptions invited, and with the modest sum of £50 the venture was launched.

As a charitable institution may legitimately be advertised, a notice announcing the opening was sent to the press and a nucleus of patients was thus obtained. For certain "puffs" which later appeared in print, together with weird sketches of patients, the committee must not be held responsible, but the press has kindly assisted us from time to time in helping the clinic to become known. In spite of appearances, from the article referred to it may be taken for granted that the place is run on strictly professional lines, with a properly constituted medical and lay committee. Moreover, letters and patients have been liberally received from the extremes of the British Isles, and many large teaching centres have sent their quota. The chief difficulty that confronted us, and one that makes the need for a multiplication of similar clinics more pronounced, is the expense and inconvenience patients experience in travelling these distances and remaining a sufficient length of time for the treatment.

A further difficulty, arising directly from this cause, is the personal element between doctor and patient. It was recognised that we should obtain better results if the same physician treated a case throughout, and this is the course adopted with the patients who reside in the neighbourhood of Liverpool, and who proceed to their homes after each session. In those who come from a distance and remain in lodgings until treatment is concluded it is realised that they must receive a sitting each day, and accordingly each doctor takes them in turn on his visit.

Our first plans were to rent a house and place a caretaker and his wife in charge, and in return for their services supply them with light, coal, and apartments. Limited means and considerations of cost were occupying our attention when we had a favourable offer from Mrs. Rose, the matron of the Maryland Street Nursing Home, who offered the needful accommodation and the services of a nurse for a sum which we considered was within our means, whilst in return she hoped to receive our recommendation to patients requiring lodgment, and for this purpose she arranged her house and terms to suit the varying classes of patient (both clinic and private) who might contemplate treatment. In this she exhibited commendable forethought, and though many patients do so use the home they nevertheless do not interfere with the carrying out of the ordinary branches of medical and surgical nursing. The rapid influx of patients soon made it necessary to move into larger premises, and we now occupy, under similar terms, with the same matron, rooms in two large houses at 25 and 27 Catherine Street, which have been structurally adapted to the combined requirements. Our methods of dealing with the patients are similar to those that I have seen in Paris.

A large room, bare of unnecessary ornamentation, carpeted, quiet, with a blue linen blind to the window, is our suggestion apartment. It is furnished with a dozen canvas chairs, not of the hammock type, but with bands to support the hollow of the back, rolls for the head, and rests for the legs. They are so disposed that a space is left between them for the physician to pass. The cases are first taken in a separate room, and brief notes are made on the case card, which the patient takes into the clinic with him for reference if needed.

The light is subdued when all are seated, and the physician passes from patient to patient, first inducing a light stage of drowsiness, or the deeper stages if he is able or desires, and afterwards making the suggestions applicable to each case.

The numbers of return visits to each patient in the course of the hour during which the session lasts naturally depends upon the number receiving treatment, and the time each is dwelt upon in the individual suggestions, but they probably average three or four.

The three physicians attend each on two days a week, for one hour in the afternoon, but probably at a later date these times may be varied, and it is hoped to undertake psycho-analysis when opportunity offers. Unless absolutely destitute the patients contribute 6d. per visit, but so many come under the head of destitution that the clinic is not self-supporting, and the funds having dwindled to nil a fresh appeal is about to be made to the public.

What we greatly desire is a regular income of £50 per annum. If we were provided with separate cubicles for each patient we could do away with what might be considered an objectionable feature of our collective treatment, for though the suggestions are inaudible to the neighbouring patient there is a sensitiveness about these poor people which often leads them to fear otherwise. Our attendances have averaged fifty per week, and all classes of functional diseases have been treated.

Cases are carefully examined before acceptance, and if considered to have an organic basis they are not treated, or are recommended to continue other treatment *pari passu*. It is hoped to give lectures, or facilities for instruction for those desiring it, as at the London Nerve Clinic.

